# Trends in modeling Biomedical Complex Systems

**DOI:** 10.1186/1471-2105-10-S12-I1

**Published:** 2009-10-15

**Authors:** Luciano Milanesi, Paolo Romano, Gastone Castellani, Daniel Remondini, Petro Liò

**Affiliations:** 1 Institute of Biomedical Technology, National Research Council, Milan, Italy; 2 Bioinformatics, National Cancer Research Institute, Genoa, Italy; 3 Physics Department of Bologna University, Galvani Center for Biocomplexity and INFN Italy; 4 Computer Laboratory, University of Cambridge, Cambridge, UK

## Abstract

In this paper we provide an introduction to the techniques for multi-scale complex biological systems, from the single bio-molecule to the cell, combining theoretical modeling, experiments, informatics tools and technologies suitable for biological and biomedical research, which are becoming increasingly multidisciplinary, multidimensional and information-driven. The most important concepts on mathematical modeling methodologies and statistical inference, bioinformatics and standards tools to investigate complex biomedical systems are discussed and the prominent literature useful to both the practitioner and the theoretician are presented.

## Introduction

**Figure 1:**
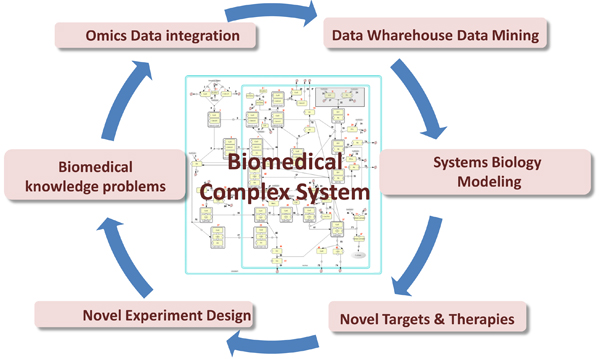
**Graphical representation of the main components for the data modeling of Biomedical Complex System**.

New "omics" technologies applied to molecular genetics analysis are producing huge amounts of raw data. Biomedical research laboratories are moving towards an environment, created through the sharing of resources, in which heterogeneous and complex health related data, such as molecular data (e.g. genomics, proteomics), cellular data (e.g. pathways), tissue data, population data (e.g. genotyping, SNP, epidemiology), as well as data generated by large scale analysis (e.g. Simulation data, Modelling, Systems Biology), are all taken into account as shown in Figure 1.

The future of biomedical scientific research will be to use massive computing data-crunching applications, data grids for distributed storage of large amounts of data and to develop new approaches to the study of the medical implications of the genome-enabled medical science. Microarrays, NMR, mass spectrometry, protein chips, gel electrophoresis data, Yeast-Two-Hybrid, QTL mapping, gene silencing, and knockout experiments are all examples of technologies that capture thousands of measures, often in single experiments.

In this review we introduce the term Biomedical Complex System, together with some examples, to characterize the complexity of current models for biological processes involved in normal and pathological states that make full use of the current available high-throughput data and potential applications are highlighted.

## Complex system application in human diseases

Human diseases result from abnormalities in an extremely complex system of molecular processes. In these processes, virtually no molecular entity acts in isolation and complexity is caused by the vast amount of dependencies between molecular and phenotype features. It is a very intuitive concept to represent such complex information as networks. The field of network theory has progressed rapidly over the last years (see [1] for a review of recent results and references) and not surprisingly, this representation of complex information has found its way into medical research [2,3]. It has been suggested that a systems based approach using network analysis could offer means to combine disease related information and to identify the most important factors for the phenotype of the disease. In particular, it has been stressed that the combination of genomic, proteomic, metabolomic and environmental factors may provide insights into pathognomonic mechanisms and lead to novel therapeutic targets. Post-genomic approaches have already contributed to the understanding of specific aspects of the disease process and to the development of diagnostic and prognostic clinical applications. Cardiovascular obesity, diabetes, autoimmune diseases, and neurodegenerative disorders are some of the disease areas that have benefited from these types of data. Such diseases are the result of disturbances at different scales in several molecular interactions and processes, which contribute to an increased susceptibility to aging, morbidity and mortality. For such diseases, a vast amount of data originating from different sources is typically available, but in common clinical practice different types of data are interpreted in isolation. It is therefore poorly understood how different factors act in synergy to cause a complex disease phenotype. The patterns of dependencies between these factors may be effectively reflected in different, connected networks that associate patients with clinical and molecular abnormalities as well as environmental determinants. This process of data integration will allow to understand better the disease phenotype and to assign patients to specific disease subtypes [4,5]. We foresee that complex diseases will prompt the development of classifiers and kernel-based approaches for clinical decision support, in which many genome-wide data sources are combined with physiological parameters within the patient domain, making use of novel modeling methodologies.

A great challenge for contemporary Molecular Medicine is the modeling, description and ultimately the comprehension of the origins of complex and multifactorial pathologies within a Systems Biology framework. Terms 'multifactorial' and 'polygenic' express the idea that multiple genes act in combination with lifestyle and environmental factors. Inheritance of polygenic traits and diseases does not fit simple patterns as in a pure Mendelian case, but there is also a strong environmental component. Many common traits, such as skin colour, height, and even intelligence, are inherently multifactorial, and also many common diseases, such as type-2 diabetes, obesity, asthma, cancers, mental retardation aging related diseases, cardiovascular diseases and obesity, tend to be multifactorial.

As an example of complex pathology, we can consider human aging. The ageing process is caused by the progressive lifetime accumulation of damages to macromolecules and cells. The capability of the body to set up a variety of molecular and cellular strategies to cope with and neutralize these damages is considered a key feature of longevity. The aging process can be influenced by several variables such as lifestyle, environment, genetics and intrinsic stochasticity. For example, transcriptional noise measurements in young and old cardiomyocytes by global mRNA amplification as well as quantification of mRNA levels in a panel of housekeeping and heart-specific genes increase in the old age compared to the young one [6]. The understanding of the aging process raises the question of stability over time of biological functions (anthagonistic pleiotropy) and of discrimination among biological and chronological age. Novel strategies may help to identify new molecular targets that can be addressed to prolong the lifespan and to improve the quality of life during aging.

Psychiatric disorders seem to particularly lend themselves for a systems based analysis approach. It is well known that schizophrenia has a strong genetic compound with concordance rates in monozygotic twins reaching approximately 50%. This increased risk is conferred by a multitude of different genes with the most important genetic polymorphisms accounting for 1% of increased risk. It seems likely that the disease is ultimately precipitated by a complex interplay of genetic predisposition and of a broad spectrum of environmental and nutritional factors (see [3] for updated references on schizophrenia and its identification as a complex network disease). In this context, epidemiological factors such as urbanicity, geographical distribution, and migration behaviour, but also maternal risk factors (such as infections, malnutrition, adverse life events during pregnancy or season of birth), have been suggested to be associated with the risk of schizophrenia onset. The relationship between these factors and the interplay with genetic determinants remains unknown, and integrated, system based investigations seem to be a promising approach to obtain deeper insights into the disease aetiology.

Metabolic syndrome is a combination of medical disorders that increase the risk of developing atherosclerosis, cardiovascular diseases, diabetes and other pathologies. It affects a significant part of population in western countries, and its prevalence increases with age. The exact patho-physiological mechanisms of metabolic syndrome are not yet completely elucidated, due to the number of involved factors, and to their interaction complexity. The most important factors are: weight, genetics, aging, and lifestyle, i.e., low physical activity and excess caloric intake. There is debate regarding whether obesity or insulin resistance (IR, i.e. the condition in which normal amounts of insulin are inadequate to produce a normal insulin response) are the cause of the metabolic syndrome or if they are consequences of a more far-reaching metabolic derangement. A number of markers of systemic inflammation, including C-reactive protein, are often increased, as are fibrinogen, interleukin 6 (IL-6), Tumor necrosis factor-alpha (TNFα) and others. Some have pointed to a variety of causes including increased uric acid levels caused by dietary fructose. In vivo and in vitro studies of insulin signalling network have provided insights into how insulin resistance can develop in some pathways, whereas insulin sensitivity is maintained in others. In a Systems Biology perspective, this phenomenon can be modelled as a form of adaptation with consequent switch between stable phenotypes. This model is supported by experimental observations showing that the pathways leading to IR contain several phosphorylation steps, and this can be sufficient to support multistability and switching among phenotypes [7].

An emerging field of Medicine is the so called Ecological Medicine, that is trying to define the health state in terms of biological community abundance, composition and type. Recent studies on gut microbiota (the intestinal bacteria heterogeneous population) show that its composition may change with pathological state and ageing. Since it is also modulated by the Immune System, it can be seen as a crucial node for determining the interactions between environment (food) and internal machinery (Immune and Metabolic system), especially for those pathologies related to both factors (see for example [8]).

## Multidisciplinary complex system theory

A number of physico-mathematical theories are dealing with systems characterized by a high number of degrees of freedom, non linear relations between parameters, high variability and stochastic behaviour. The science of Complex Biological Systems (ranging from Biochemistry, Physics, Biology, Medicine to Social Sciences) is trying to understand global behaviour and "emergent properties" (such as self-organization, robustness, formation of memory patterns, etc...) on the basis of microscopic factors like interacting molecules, complexes, organelles or whole cells, depending on the scale of the system under study. The unifying framework is that biological systems are constituted by a very high number of mutually interacting elements, that organize themselves in functional and dynamic networks, at different levels of complexity. The fundamental unit of living organisms is the cell (that constitutes a complex system in itself) representing the building block of higher levels of organization, such as tissues, organs and whole organisms. Different organisms organize themselves in societies and ecological systems, in which hundreds or even thousands of different species coexist in a dynamic equilibrium. The evolutionary history of biological systems, but also the history of single organisms, entails a series of constraints that can influence their structure and functional capacities: the role of evolution and environment can thus provide useful information about how to treat a specific problem (e.g. disease). Whereas a thermodynamic approach (in the limit of system elements going to infinity) is suitable for complex systems. Thus, the role of stochastic fluctuations has recently received a renewed interest, since the focus has moved to mesoscopic scales in which the number of interacting elements is quite small, the noise features are not so trivial (i.e. gaussian) and can drive the system towards unexpected behaviour [9,10]. Recent studies [11-13] based on fluorescence measurements onto the genome of simple bacteria (like E. coli) have showed that biological noise can be classified as intrinsic and extrinsic noise. Extrinsic fluctuations are those that affect equally gene expression in a given cell, such as variations in the number of RNA polymerases or ribosomes, that can make cell activity diverge in an initially uniform population. Intrinsic fluctuations are instead those due to the randomness inherent to transcription and translation; they should affect independently each element of the same network (e.g. gene or protein levels in the same cell) adding uncorrelated variations in the overall levels of cellular activity. A deeper understanding of the role of such noises could help in explaining the different responses of organisms to the same stressogen and pathologic input.

## Measuring and data analysis

The inherent complexity of biological systems requires suitable experimental, statistical, and computational strategies: generally speaking, biological experiments show fundamental differences from physical experiments, such as a higher and non-trivial (non-gaussian) variability, a lower number of available measurements (such as the number of points in a kinetic experiment, or simply the number of experimental repetitions) and the lack or poor availability of small-scale (single-molecule) experiments. During the last decade a large class of in vivo and in vitro measurements has been developed and/or improved to fill this gap, such as Quantitative mass-spectrometry, high throughput sequencing, proteomics, genomics, metabolomics, measurements. Imaging techniques, such as microscopy, ultrasound, CT (Computed Tomography), MRI (Magnetic Resonance Imaging), PET (Positron Emission Tomography), using molecular probes, such as quantum dots and nanoshells, are capable to produce quantitative and model-confirmatory data in a wide range of spatial and temporal intervals, from cells to organs or individuals, and from microseconds to hours. A central feature of all these imaging techniques is the ability to produce "in vivo" molecular data in a dynamic way (see [14] for a review).

A common denominator of these methodologies is the need for powerful computational analysis and sophisticated statistical elaboration. As an example, the high-throughput gene expression experiments (microarrays) have posed new classes of statistical problems, due to the huge number of statistical tests to be performed simultaneously over widely heterogeneous data, for which an accurate control of false positive/negative rate is a crucial issue. The solution of this problem has been faced in several ways, for example by developing post-hoc correction methods for the significance threshold [15,16], or by including "a priori" biological information. The observation that gene expression measurements follow a highly skewed and fat-tailed has raised the question of reconstructing the underlying network of interactions able to describe such observations [17,18].

Significance analysis at single gene level may suffer from the limited number of samples and high experimental noise, that can severely limit the power of the chosen statistical test. This problem is typically approached by applying generalized null models [19] to control the false discovery rate, or taking into account prior biological knowledge. Pathway or gene ontology analysis can provide an alternative way to enforce single-gene statistical significance, with the advantage of suggesting a clearer biological interpretation. The use of "a priori biological knowledge", as coded in pathways or ontologies, may help to detect relationships at multiple scales, grouping single gene analyses into clusters (pathways, ontologies) and super-clusters (networks of pathways, higher-order ontologies) with precise biological functions.

Among different approaches that have been proposed to identify significant gene groups, a large number is based on lists of differentially expressed genes such as GOstat [20] that compares the occurrences of each GO (Gene Ontology) term in a given list of genes with its occurrence in a reference group on the array. In the context of pathway analysis, a similar approach is used by Pathway Miner [21], which ranks pathways by means of a one-sided Fisher exact test. Other methods allow investigators the possibility to define their own gene-grouping schemes. For example, the Global Test package [22] applies a generalized linear model to determine if a user-defined group of genes is significantly related to a clinical outcome. With the Gene Set Enrichment Analysis (GSEA [23]) an investigator can test if the members of a defined gene set tend to occur towards the top or the bottom of a ranked significance list obtained from differential expression analysis. Other methods combine pathway information with Fisher exact test for 2 by 2 contingency tables and its variations [24] allowing dimensionality reduction of the problem (from 10^4 ^probes to 10^2 ^pathways) and increasing biological interpretability of the studied processes. By these methods, it is possible to consider the single-gene relevance at different levels of biological organization: groups of genes as provided by several ontology classes, pathways and metapathways. The further direction is to integrate different kinds of biological knowledge (protein-protein interaction, transcription factor network, biochemical reaction network, as well as clinical and aetiological information about the samples) into a unified framework.

## From high throughput data to modelling

Nowadays, an important area of investigation focuses on using statistical inference for mechanistic models of partially observed dynamic systems. This area represents the challenge task of combining statistical methods with models of dynamical systems. Dynamic models, usually written in forms of differential equations (DEs), describe the rate of change of a process. They are widely used in medicine, engineering, ecology and a host of other applications. One central and difficult problem is how to estimate DE parameters from noisy data. Direct approaches (such as least squares) give rise to difficulties partly because of the intrinsic definition of the mathematical model. A formal approach in specify uncertainty in systems of differential equations within a statistical inferential framework is something that mathematicians have only very recently started to consider. There is a great motivation, within the area of Computational Systems Biology, to fully define and propagate all sources of uncertainty in model-based reasoning, with reference to the genetic, biochemical and cellular mechanisms initiating and regulating fundamental biological processes. These systems are non-linear, non-steady state, and contain many unknown parameters. A single nonlinear differential equation model can describe a wide variety of behaviours including oscillations, steady states and exponential growth and decay, with relatively few parameters. Noteworthy, many DEs do not have an analytic solution, implying that a likelihood centred on the solution to the DE is full of local maxima, ridges, ripples, flat sections, and other difficult topologies. If only parts of a genetic, biochemical and cellular network can be observed directly, structural non-identifiability may then arise and manifests itself in functionally related model parameters which cannot be estimated uniquely. The challenge in implementing robust predictive analyses is that integrals over high-dimensional parameter spaces are usually involved that can neither be evaluated analytically, nor numerically in a straight-forward way. Although inference techniques, such as Maximum Likelihood, are relatively easy to implement, they suffer from drawbacks, such as not fully exploring the entire parameter space. As a solution to this problem, the generalized profiling method [25] was proposed, in which DE solutions are approximated by nonparametric functions, which are estimated by penalized smoothing with DE-defined penalty. The existing inference methods have substantial limitations upon the form of models that can be fitted and, hence, upon the nature of the scientific hypotheses that can be made and the data that can be used to evaluate them. Instead, the so called plug-and-play methods require only simulations from a model and are thus free from such restrictions. Plug-and-play inference is extremely useful when one wishes to entertain multiple working hypotheses translated into multiple mechanistic models [26]. The Bayesian methodology provides one such inferential framework, however, whilst beautifully elegant in principle, computational challenges associated with its practical instantiation are formidable [27], due to a combination of non-linear non-steady state differential equations containing many parameters in conjunction with a limited amount of data. Some currently useful computational tools are: Laplace's method of asymptotic approximation and Markov Chain Monte Carlo (MCMC) methods, including multi-level Metropolis-Hastings algorithms with tempering, the Gibbs sampler and the Hybrid Monte Carlo algorithm. Particle filter algorithms are useful for sequential Bayesian state estimation when the Kalman filter is not applicable because of non-linear dynamics and/or non-Gaussian probability models.

## Methodologies for modelling technique

What does modelling a complex system mean? From a strictly physico-mathematical point of view, it means reproducing the main features of a real system (like phase transitions and bifurcations, parametric and stochastic resonance phenomena) by means of a model with as few parameters as possible, in order to get a completely controllable system, that can possibly be treated analytically. Usually, the aim is to learn as much as possible from such a simplified problem, with the hope that the original system can be seen as a "small perturbation" of that (i.e. we are hoping to have caught the peculiar features). For a complex system, it is expected that the number of parameters and basilar elements is not too little, since, very often, the level of complexity is given by a high number of agents (composing the system) that are interacting in a non-trivial way, so that a "mean field" approach is not suitable. The search for such simplified models is, without any doubt, very useful, especially from a theoretical biology point of view, but it may not be the case for an applied biomedical problem. First, the essential ingredient of simplification, that is passing from the specific case to the more general one, is to discard as many details as possible. But in a biomedical problem we can be more interested in a particular solution of our problem, that deals with as many details as possible of the system (e.g. the features of a specific pathway involved, or the past history of the sample). The multiplicity of subclasses in which the parameter space associated to our system can be divided is the goal of our modelling, rather than something to "average out" as in a classical thermodynamic approach to a physical system. Similarly, we might be more interested in a multiparametric model that, even if analytically untractable, can be repeatedly simulated and "tuned" to a real situation, rather than in an idealized toy model that has lost any relationship with reality. Examples that go in this directions are the so called flux balance analysis (FBA) or flux optimization, in which the parameters (e.g. reaction rates) of a real system are changed in order to study how the reaction yields are affected [28-30]. In this sense, the paths to complex system modelling, from a theoretical and a more applied point of view, may run together in the beginning, but they may possibly divide along the road and take different directions. The tradeoff between the search for a model as simple as possible and an adequate description of the original complex system is more markedly present in the fields of biology and medicine (rather than in physics), because a beautiful theoretical model may be of no help if it cannot be brought back to reality. One practical task of modelling may be to help in the generalization of the results obtained from simpler organisms (that can be massively tested by experiments) to humans, by following the analogies connecting them. This actually is a common practice for inferring protein functions and interactions by looking at their (structural) similarities with proteins from simpler (and more studied) organisms. Thus, related to this task, deepening the knowledge about such analogies and their limits is of fundamental importance for fruitful biomedical achievements. This can be pursued by cleverly scanning databases, repositories and ontologies in search for common modules and structures, and a good modelling must provide hints and tools for adequate simulations of these different complex systems (e.g. in their dynamics of response to stimuli [31,32]).

A classical case study in biophysics is the induction and maintenance of memory in biological systems (from small genetic circuits to whole cells as neurons). The mathematical formulation of this problem is related to the concept of bistability, both in a deterministic and in a stochastic formulation. Deterministic bistability is typically governed by feed-back, auto-catalysis and non linear interactions, and can be appreciated by stability and robustness analysis. Stochastic bistability is more subtle, crucially related to noise level as well as to the real size of the systems (e.g. the number of proteins participating in a reaction). An approach that has received renewed attention, is based on the so called Chemical Master Equation (CME) that describes the temporal evolution of the probability of having a given number of molecules for each chemical species involved. The discrete probabilistic approach, as with CME, is attractive because it ensures the correct physical interpretation of fluctuations in the presence of a small number of reacting elements (as compared to continuum approaches as Langevin and Fokker-Planck formalism [33]) and because it provides an unitary formulation for many biological processes, from chemical reactions to ion channel kinetics. The CME theory can be related to predictions on the noise levels in selected biological processes, as for example during transcription and translation [34,8]. In particular, the observation that mRNA is produced in bursts varying in size and time has led to the development of new models capable of better explaining the distributions of synthesized products [35].

## Computing and standards for large scale simulation

Due to large data sets and accompanied large number of parameters being produced by high throughput techniques such as next-generation sequencing able to accelerate the entire process from sample preparation to data analysis, there is a growing usage of high performance computers based on clustering technologies and high performance distributed platforms. A first approach to scalable computer infrastructure has been the use of large supercomputer cluster following by the introduction of the grid computing; a more recent one is the cloud computing. Grid infrastructures are based on a distributed computing model where easy access to large geographical computing and data management resources is provided to large multidisciplinary Virtual Organizations (VOs). The distributed High Performance Computer (HPC) is considered the way to realize the concept of virtual places where scientists and researchers work together to solve complex problems, despite their geographic and organizational boundaries. Cloud computing is defined and characterized by massive scalability and Internet-driven economics realised as a pool of virtualized computer resources. A Cloud Computing platform supports redundant, self-recovering, highly scalable programming models that allow workloads to recover from many unavoidable hardware/software failures, as well as to monitor resources use in real time for providing physical and virtual servers on which the applications can run. In a Cloud Computing platform software is migrating from the desktop into the "clouds" of the Internet, promising users anytime, anywhere access to their software and data.

### Characteristics of biological data sources

A huge amount of biological and medical information is now publicly available. Emerging knowledge domains, tightly linked to systems biology, like interaction networks and metabolic pathways, are contributing with even huge amounts of data. Information in secondary databases represents an essential resource for researchers since they target special research interests. Many databanks are created and maintained by small groups or even by single researchers. As a result of this diffused and uncoordinated development, data is spread over hundreds of Internet sites and included in a high number of heterogeneous databases, the majority of which are of a great interest for systems biology, where it is stored using different database management systems and data structures. There are little common information sets and the semantics of data, i.e. the actual meaning associated to each piece of data, is left to the developers. It can therefore be different, even when using same or similar names, thus leading to potential confusion. User interfaces and query methods are also different and searching, retrieving and integrating information may become very difficult.

### Data integration

One of the main issues in systems biology data management is data integration in order to represent the global view of biological information. The data management involves retrieval of information from multiple databases and the execution of large scale data analysis. Data integration can be best achieved when the information and desired analysis are stable in time and based on standardization of data models and formats. In biology the domain's knowledge changes very quickly and the complexity of information makes it difficult to design complex data models. Integrating biological information in a distributed, heterogeneous environment requires expandable and adaptable technologies and tools that are able, at the same time, to cope with the heterogeneity of data sources and to select and manage properly the right information, i.e. by recognizing its semantics.

Among current Information and Communication Technologies (ICT), the eXtensible Markup Language (XML) [36] together with XML based biologically oriented languages, and Semantic tools, like ontologies, are the most interesting ones in view of the achievement of a standardized environment for systems biology. A Markup Language (ML) is a mechanism aimed at defining parts of a document (i.e. data) by surrounding it with a start and an end tag. XML specification defines a way to add markup (tags) to documents and thus assign meanings to data explicitly. A set of tags and their relationships defines an XML language and constitutes a namespace, the context where those tags are valid. XML languages are defined by using Document Type Definitions (DTDs) or XML Schemas [37].

Many XML languages have been created for biology, more than can be reviewed here. For the reasons of their adoption and a short list, see [38-40]. They range from the basic one, e.g. for the storage of databanks information in alternative formats that can improve traditional flat-file management, and for the description and archiving of results of main analysis tools, to the most complex, like those used in specialized knowledge domains (e.g., the Polymorphism Markup Language (PML) [41] that has been developed as a common data exchange format to overcome the heterogeneity of SNPs databases.

XML languages can also support data interchange. In order to simplify interoperation between bioinformatics tools, the HOBIT XML schemas, that refers to some bioinformatics data types (sequence, RNA structure and alignment), and the BioDOM software library for their management were developed [42].

An ontology is the "specification of conceptualization" in a given domain of interest. It consists of a set of concepts in that specific domain, expressed by using a controlled vocabulary, and of the relationships among these concepts. Ontologies can add semantic metadata to the resources, improve data accessibility and support integrated searches. Many biomedical ontologies have been, or are being, developed, mainly in the context of the Open Biomedical Ontologies (OBO) initiative [43].

### Standards for systems biology

Many reviews have already been published on standardization of data and tools in support of systems biology development and research. We here refer to them, due to their completeness and authoritativeness.

Brazma et al published an accurate review in 2006 [44]. They pointed out the main objectives of standardization in life sciences, gave a classification of existing standards and produced extended and accurate lists of acronyms, definitions, URLs. Their classification is based on a table where rows represent three areas of systems biology (biological knowledge, evidence produced by technologies, general frameworks), and columns represent the four steps of standardization that they define in the review, namely informal semantics, formal semantics, formal syntax and tools.

Strömbäck et al [45] and Wierling et al [46] published reviews where the focus was posed on tools, data standards, the role of XML languages for data exchange and how ontologies are used to develop new formats, thus constituting an essential component of standardization.

The Systems Biology Markup Language (SBML) [47,48] is defined as "a computer-readable format for representing models of biochemical reaction networks". The same objectives are driving the development of the Cell System Markup Language (CSML) [49] with the aimed to visualizing, modelling and simulating biopathways. The most used data standards are summarize in Table 1.

**Table 1:** Standards for systems biology, grouped by type

**Minimum Information (see also the MIBBI initiative **[58])		
MIAME	Microarray Experiment	
MIAPE	Proteomics Experiment	
MIRIAM	Annotation of biochemical Models	
MIACA	Cellular Assay (MIACA)	

**Ontologies (see also the OBO Foundry **[43])		

GO	Gene Ontology	
MO	Microarray Ontology	
PW	Pathway ontology	
PSI-MI	Protein-protein interaction	
SBO	Systems Biology	
FuGO	Functional Genomics Investigation	

**XML Languages**		

MAGE-ML	Microarray Gene Expression ML	
HUP-ML	Human Proteome ML	
mzXML	Mass spectrometry data ML	
SBML	Systems Biology ML	
CSML	Cell System ML	
CellML	Cell ML	
BioPAX	Biological Pathways Exchange	

### Database supporting systems biology research

Systems biology research depends on availability of well structured data sources. Moreover, data is rarely integrated in databases that alone can support research in even the smallest biological domains. Eils et al suggest an "integrative database for systems biology", defined as a "data warehouse system" supporting all activities of a systems biology project [50]. The system would consist of three modules, one for each of the main involved data subsets: i) experimental data, ii) components and reactions of biological systems, and iii) mathematical models. Both functional models and simulations are stored by using the SBML format, thus emphasizing the role and the relevance of standardization in this field.

Many databases are relevant for systems biology and a more extended list with emphasis on databases on models and pathways is presented in Table 2. It is implicit that most of the primary databases, from gene and protein interaction, are also of interest for systems biology.

**Table 2:** Some of the most interesting databases for systems biology

KEGG	Kyoto Encyclopedia of Genes and Genomes Incudes data on genes and proteins, endogenous and exogenous ligands, diagrams of interaction and reaction networks, and hierarchies and relationships of various biological objects	
BRENDA	The Comprehensive Enzyme Information System Information on enzymes and their function	

BioCyc	Collection of Pathway/Genome Databases Derived from literature (EcoCyc, MetaCyc) or computed (some 500 databases, maìnly for bacteria, but also incl. HumanCyc and MouseCyc, about 20 of which are partially corrected by volunteers)	

CSNdb	Cell Signaling Networks Database Information on molecules, sequences, structures, functions, and reactions transferring cellular signals in human cells	

BioBase	Many commercial databases offered on-line with reduced functionalities Includes TRANSFAC (transcription factors and their binding sites and regulated genes) and TRANSPATH (molecules involved in signal transduction pathways and related reactions)	

BioModels	A Database of Annotated Published Models Includes mathematical models published in literature. Models are annotated and linked to esternal databases and to literature.	

BioUML	BioUML is framework for systems biology. It spans the comprehensive range of capabilities including access to databases with experimental data, tools for formalized description of biological systems structure and functioning, as well as tools for their visualization and simulations.	

CCDB	The CCDB is a cell cycle database Is an integrated data warehouse database for systems biology modelling and cell cycle analysis	

PID	The Pathway Interaction Database A curated database of biomolecular interactions and key cellular processes assembled into signaling pathways focused on human data. Human pathways from Reactome are also included. PID is a collaboration between the NCI and Nature Publishing Group.	

Reactome	A curated knowledgebase of biological pathways Information is authored by experts and curated by internal staff. It includes links to external data sources, like KEGG Compound, ChEBI, PubMed, and GO.	

BioModels is a database of annotated computational models [51], manually curated at the European Bioinformatics Institute (EBI) as part of the broader BioModels.net initiative [52]. The BioCyc Database Collection [53] is a collection of Pathway/Genome Databases, either derived from literature or computed, including more than 500 species, mainly bacteria, but also including homo sapiens and mus musculus. MetaCyc is instead a database of non-redundant, experimentally elucidated, metabolic pathways [54].

### A methodology for the development of new tools for systems biology

The following methodology for the development of new tools for systems biology is meant to implement ways for sharing data models and definitions based on common data interchange formats [55]:

• XML schemas can be used for the creation of common models of biological information,

• XML based languages can be adopted for data storage, representation and exchange,

• Web Services can be made available for the interoperability of software,

• ontologies can semantically support Web Services discovery, selection and interoperation,

• Workflow Management Systems can then be used to implement automated processes.

Although this methodology can be seen very difficult to be implemented, the Microarray Gene Expression Data Group (MGED) [56] initiative, lead along the above lines, can instead be seen as a success story. MGED is an international society of biologists, computer scientists, and data analysts that aim to facilitate the sharing of microarray data. This initiative was devoted to the creation of a common data structure for communicating microarray based gene-expression (MAGE) data. This activity started by defining the Minimum Information About a Microarray Experiment (MIAME) data set. MIAME describes the data that is needed to interpret unambiguously results of any experiment and potentially reproduce it [57]. MIAME includes raw and normalised data for each hybridisation in the study, annotations of the sample and of the array, and other related information. In order to improve specification of MIAME information, and therefore its accessibility, a data exchange model (MAGE-OM) and related data formats were then defined. Formats are specified as spreadsheets (MAGE-TAB) and as an XML language (MAGE-ML). In addition, the MGED Ontology was developed for the description of key concepts. A software toolkit (MAGE-STK) was finally developed to facilitate the adoption of MAGE-OM and MAGE-ML.

Along these lines, many 'Minimum Information' datasets have been defined in other biological domains. Currently, the Minimum Information for Biological and Biomedical Investigations (MIBBI) initiative lists some 30 such specifications [58]. This is an extremely good starting point towards a widespread adoption of above methodology.

## Methodology for the description of complex biochemical systems

In the last decade, different computing paradigms and modelling frameworks for the description and simulation of biochemical systems have been introduce to describe the complex biological system. Usually the parameter values are generally unknown or uncertain due to the lack of measurements, experimental errors and biological variability and this is a great problem in the development of cellular models [59]. In the following paragraph we introduce some of the most important methodologies used to describe the complex biochemical systems:

### • Nonlinear Ordinary differential equations (ODE)

Nonlinear ordinary differential equations represent an important approach used to describe cellular dynamical properties when it is possible to consider component diffusions instantaneous and concentrations sufficiently high [60]. Considering the cell cycle, ODEs are widely used to describe the dynamics of its regulations and all the related published models are stored in the Cell Cycle Database [61]. Moreover, a specific set of parameters values describe a model in a particular physiological state and in a peculiar species. The core regulation of the cell cycle is widely conserved across different species but time scales can vary from minutes to hours and the key elements interactions involved in processes typically vary across species [62].

### • Membrane system (P system)

Membrane systems, also known as P systems have been introduced in [63], are computation models as a class of unconventional computing devices of distributed, parallel and nondeterministic type, inspired by the compartmental structure and the functioning of living cells. In order to define a basic P system, three main parts need to be introduced: the membrane structure, the objects and the rules. For a complete and extensive overview of P systems, we refer the reader to [64] and to the P Systems Web Page .

### • Tissue system (tP system)

tP systems have been introduce to describe a tissue like architecture, where cells are placed in the nodes of a directed graph, and objects are communicated along the edges of the graph. These communication channels are called synapses. Moreover, the communication of objects is achieved both in a replicative and non-replicative manner, that is, the objects are sent to all the adjacent cells or to only one adjacent cell, respectively. The variants of tP systems considered in the literature essentially differ in the mechanisms used to communicate objects between cells. [64,65].

### • Stochastic simulation technique (τ-DD system)

The definition of the stochastic simulation technique called τ-DD [66], where the probabilities are associated to the rules, following the method introduced by Gillespie in [67]. The aim of τ-DD is to extend the single-volume algorithm of tau-leaping [68], in order to simulate multi-volume systems, where the distinct volumes are arranged according to a specified hierarchy. The τ-DD approach is designed to share a common time increment among all the membranes, used to accurately extract the rules that will be executed in each compartment (at each step). This improvement is achieved using, inside the membranes of τ-DD, a modified tau-leaping algorithm, which gives the possibility to simulate the time evolution of every volume as well as that of the entire system.

## Conclusion

In this paper we reviewed some of principal concepts that, in our opinion, will characterize the next future of Systems Biology and of interdisciplinary research in biomedicine. A key point emerging from this review is the characterization and definition of complex biomedical system. The notion of complexity, although still elusive, is giving new tools for the interpretation of Biology and Medicine. A new interface between Medicine and Biology is emerging with the contribution of other sciences such as Physics, Engineering and Mathematics. As a consequence, new conceptual frameworks are taking place in biomedicine, and it is becoming clear that it is no more possible to neglect properties of biomedical systems arising from small-scale elements like noise, fluctuations, and global properties, such as integrated responses at the whole organism level. Within this scenario, a central role is played by new techniques for producing and analyzing data that are giving a detailed picture of the mechanisms governing biological systems, including humans, that support the concept of obtaining a personalized medicine. Advanced computer simulation techniques are having an increasing diffusion in the biomedical disciplines, and are providing new methodologies for the prediction of their behaviour. These methodologies range from deterministic to stochastic algorithms, and are supported by new generations of hardware that allow huge data storage capability and computational power. The availability of data storage and standard communication protocols has fuelled the appearance of a series of public database and Web Services, in which it is possible to retrieve a wide class of biological information. Finally, the conceptual framework of Systems Biology and the definition of Complex Biomedical System, are giving a new interpretation of complex pathologies and therapeutic approaches. These modeling approaches aim to bridge the 'translational gap' between basic and clinical research towards translational medicine.

## Glossary

*Bifurcation*: a change in the properties of the stable states of a system described by a mathematical function, e.g. when the average value of a protein passes from a single possible value (one stable state) to a high or low possible value (two stable states)

*Emergent properties*: global properties of a system resulting from simpler interaction of its elements, rather than being specifically encoded in it

*False discovery rate*: statistical method to control (or at least to estimate) the expected number of false positives (e.g. cases called different from the null hypothesis when they are not) when applying multiple testing (i.e. many statistical tests in parallel, e.g. while checking for statistically significant differential expression of thousand of genes).

*Fisher's exact test*: a statistical test to check for nonrandom association between two variables

*Generalized linear model*: a generalization of linear regression between two groups of variables that may allow for nonlinear relationships

*Maximum Likelihood (ML)*: The likelihood (L_H) of a hypothesis (H) is equal to the probability of observing the data if that hypothesis were correct. The statistical method of maximum likelihood (ML) chooses amongst hypotheses by selecting the one which maximizes the likelihood; that is, which renders the data the most plausible.

*Markov process*: A mathematical model of infrequent changes of (discrete) states over time, in which future events occur by chance and depend only on the current state, and not on the history of how that state was reached.

*Null model*: the "null hypothesis" for a statistical test describes the typical background properties of the model that should be contradicted in case of significant deviations from it.

*MonteCarlo Markov Chain*: Standard MCMC uses a Markov Chain where a new state is proposed, then with some probability, the proposed state is accepted or the previous state is maintained. After a long time of continuing this process, (under some conditions) states visited by the Markov Chain approximate a sample from the posterior density of model parameters given the data.

*Plug and play statistics Statistical methods*: are plug-and-play if they require simulation from a dynamic model but not explicit likelihood ratios.

*Phase transition*: a macroscopic change in system properties that result in discountinuous variations in some observed variables (e.g. the change from liquid to gas as a function of temperature and pressure changes).

*Stochastic fluctuations*: changes in time (and space) of an observed variable (e.g. the expression of a protein) due to random perturbations (e.g. in the degradation or synthesis processes).

*Thermodynamic approach*: a physical approach to the study of large systems in which the properties of the single elements (in the limit of the number of elements going to infinity) are averaged out but macroscopic features are kept (e.g. the average pressure of a gas obtained as an average of the single molecular impacts on the container surface).

## Competing interests

The authors declare that they have no competing interests.
